# Exploring the Correlation Between Health Literacy and Knowledge of Cervical Cancer and Radiotherapy Among Japanese Women: A Web-Based Survey

**DOI:** 10.1007/s13187-024-02432-x

**Published:** 2024-05-29

**Authors:** Masanari Minamitani, Kosuke Morishima, Atsuto Katano, Shingo Ohira, Keiichi Nakagawa

**Affiliations:** 1 Department of Comprehensive Radiation Oncology, The University of Tokyo, 7-3-1 Hongo Bunkyo-Ku, Tokyo, 113-0033 Japan; 2 Department of Radiology, The University of Tokyo Hospital, 7-3-1 Hongo, Bunkyo-Ku, Tokyo, 113-8655 Japan

**Keywords:** Health literacy, Cervical cancer, Raidotherapy, Cancer screening, HPV vaccination

## Abstract

Health literacy (HL) plays a vital role in an individual’s ability to make informed health decisions. Japan faces several challenges in cervical cancer control, including low human papillomavirus (HPV) vaccination and screening rates, underutilization of radiotherapy, and limited HL. This study explored the association between HL and knowledge of cervical cancer and radiotherapy, particularly among young Japanese women. We conducted a web-based survey among users of LunaLuna, a popular women’s healthcare application, to assess their HL and knowledge about cervical cancer and radiotherapy through a 46-question survey. We compared three groups in terms of HL (inadequate, problematic, and sufficient & excellent). Multiple regression analysis was used to identify factors associated with knowledge. In total, 1468 respondents were included in this study. HL was positively correlated with knowledge scores (inadequate: 51.8%; problematic: 56.3%; sufficient & excellent: 60%). Participants displayed relatively low accuracy for treatment-related questions. Higher HL (*β* = 0.15, *p* < 0.01), education (*β* =  − 0.11, *p* < 0.01), cervical cancer screening (*β* =  − 0.11, *p* < 0.01), income (*β* = 0.09, *p* < 0.01), and employment (*β* =  − 0.06, *p* = 0.04) were significant factors affecting knowledge of cervical cancer and radiotherapy. Our findings underscore the pivotal role of HL in promoting cervical cancer prevention and providing a better understanding of radiotherapy. Despite factors such as age, education, and history of cervical cancer screening, HL showed the strongest association with knowledge of cervical cancer and radiotherapy. The enhancement of HL and knowledge dissemination may be critical for promoting cervical cancer prevention and radiotherapy in Japan.

**Supplementary Information:**

The online version contains supplementary material available at 10.1007/s13187-024-02432-x.

## Introduction

Health literacy (HL) is defined as “the degree to which individuals have the ability to find, understand, and use information and services to inform health-related decisions and actions for themselves and others” [1]. It is often linked to their socioeconomic background [2]. Despite Japan’s high life expectancy, its population’s HL level is reportedly lower than that of other nations [3, 4]. Limited HL may negatively affect an individual’s ability to perform health-related tasks, potentially leading to increased mortality rates, reduced cancer screening and vaccination, and inappropriate decision-making processes [2].

In 2020, the World Health Organization implemented a global initiative to accelerate the elimination of cervical cancer using a triple-intervention strategy involving vaccination, screening, and treatment [5]. Japan urgently needs to devise efficient strategies to combat cervical cancer. Cervical cancer-related fatalities are expected to decline in other high-income countries, but increase in Japan [6]. Low rates of human papillomavirus (HPV) vaccination and cervical cancer screening are considered significant contributing factors [7, 8]. The Japanese government approved bivalent and quadrivalent vaccines in 2009 and 2011, respectively [7]. In 2010, a national HPV-subsidizing program for girls aged 12–16 was initiated, and subsequently, in 2013, a national HPV immunization program was launched, leading to the provision of free vaccines [7]. However, media reports of adverse events rapidly ensued, even though the Global Advisory Committee of Vaccine Safety had validated the vaccine’s safety [7]. Consequently, the Ministry of Health, Labour and Welfare (MHLW) withdrew all proactive recommendations for the cervical cancer vaccine [7], resulting in vaccination coverage plummeting to < 1% [7]. Nevertheless, in 2022, proactive vaccination recommendations were reinstated, and catch-up vaccinations for girls who missed their opportunity during suspension were initiated [9]. The participation rate in cervical cancer screening in Japan significantly lags behind that in high-income countries, at only 40% compared with their 70–80% [8]. Other government-recommended cancer screening programs targeting gastric, lung, colorectal, and breast cancers also report low screening rates. A national survey identified time constraints (29%) and overconfidence in personal health (25%) as the primary reasons for the lack of participation [10]. Historically, Japan’s treatment approach for cervical cancer has deviated from international standards, with less emphasis on radiotherapy in locally advanced cases [11]. The delayed availability of radiotherapy in Japanese gynecology has led to a preference for surgical resection as the primary treatment [12]. In addition, deviations from guidelines, such as underutilization of “concurrent chemoradiotherapy (CCRT) as the first-line treatment for stage III or IVA” and “post-operative CCRT for pN1,” were observed [13]. Limited HL may contribute to suboptimal rates of both cervical cancer screening and HPV vaccination, as well as the infrequent selection of radiotherapy over surgery.

To address these issues, it is crucial to enhance awareness and knowledge of cervical cancer and radiotherapy, particularly among young women in Japan. Previous research has suggested a positive correlation between HL and cancer knowledge [14]. This study aimed to examine the potential relationships between HL and knowledge of cervical cancer and radiotherapy.

## Methods

The survey was conducted using LunaLuna (https://www.mti.co.jp/eng/?page_id=2755), a commercial women’s healthcare service offered as a mobile phone application. LunaLuna provides users with menstrual cycle, fertility, and ovulation predictions. It also offers relevant healthcare information based on user-submitted records. As of 2021, LunaLuna stood as the most popular mobile healthcare services for women in Japan, boasting over 17 million downloads. Further demographic insights from MTI Ltd., which manages LunaLuna, reveal that 40% of the users are in their 20 s, 30% are in their 30 s, and 15% are in their 40 s. The geographical distribution of the users reflects the general population spread across Japan, with a balanced representation in terms of age and residential areas. The platform has also been used for user surveys and research.

The “Survey on Health Literacy and Radiotherapy and Cervical Cancer Awareness” was conducted within the LunaLuna service and the study participants were recruited. After being informed of the purpose of the study, participants were invited to take a 46-question survey. This survey included questions on sociodemographic background, vaccination and screening history, the short version of the European Health Literacy Survey Questionnaire (HLS-Q12), and knowledge about cervical cancer and radiotherapy [4, 15]. The Japanese recommendation regarding the target age and frequency of screening for cervical cancer is cytology (pap smear) every 2 years for women aged over 20 years [8]. Questions regarding radiotherapy mirrored those used in our previous study [16]. No financial incentives were provided for participation. This study was approved by the Research Ethics Committee of the Faculty of Medicine at the University of Tokyo (2022136NI).

The inclusion criteria required participants to be female, aged ≥ 20 years, and consenting to the purpose of the study. The exclusion criteria were non-responsiveness to questions on cancer history and HPV vaccination, failure to answer > 20% of the HLS-Q12, and non-response to at least one question on cervical cancer and radiotherapy.

HL was assessed using the Japanese translation of the HLS-Q12, a short version of a widely recognized global scale, with scores ranging from 0 to 50 [4, 15]. Participants’ HL was classified as “inadequate (0–25),” “problematic (26–33),” or “sufficient (34–42) & excellent (43–50)” based on their HLS-Q12 scores [4, 15]. Participants’ background characteristics and knowledge of cervical cancer and radiotherapy were compared across these HL categories. The Bonferroni method was used for multiple comparisons. Multiple regression analysis was used to identify factors associated with knowledge. The dependent variable was the total percentage of correctly answered questions, while the independent variables included age, education, occupation, income, and personal and family cancer history, including radiotherapy, HPV vaccination, cervical cancer screening, and HL categories. Multicollinearity was checked before conducting the stepwise regression analyses. All statistical analyses were performed using SPSS version 27 with the significance level set at 5%.

## Results

The survey was conducted between September 26th and October 3rd, 2022. Of the 2053 respondents, 1468 were analyzed, after exclusion of the others based on factors such as sex (8 males, 31 non-responsive), age (97 were 19 years old, 8 non-responsive), history of cancer diagnosis (45 unknown, 34 non-responsive), history of vaccination (17 unknown, 1 non-responsive), HLS-Q12 (249 with > 20% unknown or non-responsive answers), and incomplete responses to knowledge questions (249 who skipped at least one question).

Table 1 presents the participants’ characteristics. More than half of the participants were categorized into the inadequate HL group. No significant differences were observed between groups. The majority had no personal experience with cancer or radiotherapy and < 20% had received HPV vaccinations. Vaccination coverage by age is shown in Supplementary Fig. 1, with > 40% of the participants aged 22–28 years reporting that they had been vaccinated. Only one-third of participants complied with the recommended schedule for cervical cancer screening. Among those who were aware of cervical cancer screening, but had not undergone it, the primary deterrents were fear of pain, time constraints, and financial burden (Supplementary Table 1).

**Table 1:** Participants’ characteristics

	Whole		Inadequate		Problematic		Sufficient & excellent		*p* value
	(*N* = 1468)		(*N* = 844)		(*N* = 405)		(*N* = 219)		
	*N*	%	*N*	%	*N*	%	*N*	%	
Age group									0.42
20–29	312	21%	170	20%	93	23%	49	22%	
30–39	649	44%	393	47%	167	41%	89	41%	
40–49	445	30%	248	29%	129	32%	68	31%	
≥ 50	62	4%	33	4%	16	4%	13	6%	
Education									0.13
Undergraduate, postgraduate, medicine, or welfare	810	55%	447	53%	233	58%	130	59%	
Some high school or technical education (excluding medicine and welfare)	658	45%	397	47%	172	42%	89	41%	
Employment									0.37
Full-time employee, self-employed	738	50%	414	49%	205	51%	119	54%	
Others	730	50%	430	51%	200	49%	100	46%	
Income									0.09
≤ JPY 4,000,000 or no answer	1169	80%	685	81%	321	79%	163	74%	
> JPY 4,000,000	299	20%	159	19%	84	21%	56	26%	
Cancer history									0.98
Yes	50	3%	29	3%	14	3%	7	3%	
No	1418	97%	815	97%	391	97%	212	97%	
Radiotherapy experience									0.45
Yes	9	1%	6	1%	3	1%	0	0%	
No	1459	99%	838	99%	402	99%	219	100%	
Radiotherapy experience of family									0.51
Yes	284	19%	167	20%	71	18%	46	21%	
No	1184	81%	677	80%	334	82%	173	79%	
HPV vaccination									0.19
Yes	235	16%	124	15%	76	19%	35	16%	
No	1233	84%	720	85%	329	81%	184	84%	
Cervical cancer screening experience									0.38
Yes (following the recommendations^※^)	409	28%	219	26%	120	30%	70	32%	
Yes (not following the recommendations^※^)	747	51%	444	53%	199	49%	104	47%	
No	312	21%	181	21%	86	21%	45	21%	

*JPY* Japanese yen, *HPV* human papillomavirus

^※^The recommendation of cervical cancer screening is cytology (pap smear) every 2 years for women aged over 20 years

Table 2 displays the participants’ accuracy rates for the questions on cervical cancer and radiotherapy. The average accuracy rate for all 17 questions was as follows: “inadequate” (51.8%), “problematic” (56.3%), “sufficient & excellent” (60%). In multiple comparisons, a significant difference was observed across all responses (*p* < 0.01). The participants displayed relatively low accuracy rates for treatment-related questions on cervical cancer and the effectiveness of radiotherapy (accuracy rate: Questions C5, 29%; C6, 25%; C7, 34%; R7, 16%).

**Table 2:** Comparison of correct answer rates to cervical cancer- and radiotherapy-related questions

Number	Question	Answer	Whole		Inadequate		Problematic		Sufficient & excellent		*p* value
			(*N* = 1468)		(*N* = 844)		(*N* = 405)		(*N* = 219)		
			*N*	%	*N*	%	*N*	%	*N*	%	
C1	There are two types of cancer in the uterus: cervical cancer, which forms at the cervix, and uterine cancer, which forms in the body of the uterus	Correct	1235	84%	690	82%	347	86%	198	90%	0.01
C2	Uterine cancer is caused by some virus and could be prevented by vaccination	Incorrect	556	38%	293	35%	166	41%	97	44%	0.00
C3	The cervical cancer screening rate in Japan is comparatively lower than that in Europe and the United States	Correct	1164	79%	660	78%	329	81%	175	80%	0.32
C4	To treat early-stage cervical cancer, patients do not necessarily require the removal of their uterus	Correct	1265	86%	704	83%	359	89%	202	92%	0.00
C5	Most cervical cancers can be completely cured with radiotherapy or chemoradiotherapy	Correct	432	29%	233	28%	126	31%	73	33%	0.04
C6	Most uterine cancers can be completely cured with radiotherapy or chemoradiotherapy	Incorrect	368	25%	203	24%	91	22%	74	34%	0.00
C7	The cure rate for most cervical cancers is comparable between surgery and radiotherapy	Correct	492	34%	258	31%	138	34%	96	44%	< 0.01
R1	Cancer cells appear every day, even in healthy people	Correct	1167	79%	633	75%	341	84%	193	88%	< 0.01
R2	Cancer cells evade immune cell attacks and grow	Correct	838	57%	451	53%	251	62%	136	62%	< 0.01
R3	A medical physicist is a technician who irradiates patients under the doctor’s order	Incorrect	494	34%	276	33%	137	34%	81	37%	0.14
R4	Patients feel the irradiated lesion hot during radiotherapy	Incorrect	582	40%	319	38%	163	40%	100	46%	0.20
R5	Radiation therapy damages not only cancer cells but also normal cells	Correct	1005	68%	559	66%	287	71%	159	73%	0.02
R6	Radiotherapy delivery systems can adjust the beam shape to fit the shape of the cancer	Correct	750	51%	399	47%	221	55%	130	59%	0.00
R7	Radiotherapy cannot completely cure cancer patients	Incorrect	240	16%	132	16%	62	15%	46	21%	0.25
R8	Most patients can undergo radiation therapy on an outpatient schedule	Correct	925	63%	508	60%	266	66%	151	69%	0.10
R9	Most radiotherapy treatments are expensive and not covered by health insurance	Incorrect	693	47%	352	42%	209	52%	132	60%	< 0.01
R10	Patients don’t have the right to decide their treatments, but their doctors have it	Incorrect	1352	92%	765	91%	385	95%	202	92%	0.01

Among Japanese women by the level of health literacy (*N* = 1468)

Table 3 presents the association between the accuracy rates and participant characteristics. Higher HL (*β* = 0.15, *p* < 0.01), higher or medicine-related education (*β* =  − 0.11, *p* < 0.01), cervical cancer screening adherence (*β* =  − 0.11, *p* < 0.01), higher income (*β* = 0.09, *p* < 0.01), and employment status (*β* =  − 0.06, *p* = 0.04) were significant factors.

**Table 3:** Multiple regression analysis with the correct answer rates to cervical cancer- and radiotherapy-related questions

	*B*	SE	*β*	*p* value
Income	4.31	1.36	0.09	< 0.01
Employment	− 2.25	1.08	− 0.06	0.037
Education	− 4.27	1.01	− 0.11	< 0.01
Cervical cancer screening experience	− 2.97	0.69	− 0.11	< 0.01
Health literacy group	3.86	0.65	0.15	< 0.01
*R*^2^	0.084			
*adj. R*^2^	0.081			

Abbreviations: *SE* standard error, *B* regression coefficient, *β* standardized regression coefficient

## Discussion

This study explored the association between HL and knowledge of cervical cancer and radiotherapy, among young Japanese women. Our web-based survey demonstrated a positive association between HL and knowledge of cervical cancer and radiotherapy. A previous review clarified that HL encompasses three integral elements: knowledge of health, the ability to process and utilize health-related information, and the ability to maintain health [17]. Health knowledge comprises four facets: medicine, health, health systems, and science [17]. Knowledge of medicine includes the understanding of drugs, treatments, and diseases within a medical context [17]. However, it does not refer to specific diseases or medical practices. The unique contribution of this study lies in its exploration of the relationship between HL and knowledge of cervical cancer and radiotherapy, two important cancer-related issues among young Japanese women.

Cervical cancer control in Japan is beset by problems such as low rates of vaccination and screening, and underutilization of radiotherapy. Regarding the current vaccination situation, half of the mothers were unsure about the safety of HPV vaccines and expressed concern about severe adverse events due to unverified reports [7, 18]. This was reflected in the low percentage (38%) of correct responses to vaccine-related questions. In terms of screening, the most commonly cited reasons for avoiding screening, not just for cervical cancer, in a 2019 survey were lack of time (29%), confidence in one’s health (25%), and the assumption that one can consult a healthcare provider when needed (23%) [10]. Our survey revealed that women who forgo cervical cancer screening may have heightened anxiety about the pain associated with cervical screening. Regarding treatments, Japan has many problems such as deviation from guidelines and lack of utilization of radiotherapy [13]. We believe this is partially attributable to the Japanese patients’ decision-making style with a tendency toward paternalism, a lack of shared decision-making, and a surgical-oriented treatment policy [11, 19]. Empowering healthcare seekers to participate more actively in health-related decisions can help improve health inequalities [20]. Correct knowledge may be necessary for appropriate treatment decisions, but our results showed that the correct answer rate for treatment was lower than that for the other questions. The acquisition of basic knowledge about cancer treatment may be useful before it is necessary.

A correlation between higher literacy levels and greater knowledge was also evident in the context of radiotherapy. The questions about cancer and radiotherapy knowledge were identical to those used in our previous study in which we surveyed teachers using a web-based questionnaire to assess the effectiveness of radiotherapy video materials [16]. The overall average correct answer rate in this study (55%) was higher than that in our previous study (47%). This difference might be due to the fact that women in the Japanese population tend to have better knowledge about cancer than men [21]. Our study revealed a disappointingly low positive response rate (16%) to the curative potential of radiotherapy. This reflects the common perception that radiotherapy is less curative than surgery [22].

Multiple regression analysis identified HL, education, cervical cancer screening, income, and job experience as factors associated with the correct response rate. Notably, those with higher correct answer rates were more likely to be full-time employees or self-employed. Our previous research reported no significant differences in cancer knowledge based on education, income, or employment status, but found a correlation between willingness to undergo screening and knowledge [21]. While age alone did not significantly affect knowledge outcomes, the stratified analysis revealed that higher income and enhanced health literacy were significantly correlated with the percentage of correct responses in all age groups (Supplementary Table 2a–c). It is interesting to note that the influence of education was most pronounced among participants in their 20 s, suggesting its critical role in building knowledge at earlier ages in life. For participants in their 30 s, health literacy was the dominant factor, highlighting its increasing importance with age. For those aged 40 and older, previous experience of cervical screening was significantly associated with knowledge, possibly reflecting a cumulative effect of ongoing engagement with health issues over time. Conclusively, the coefficient of HL was the largest, indicating that HL was most strongly associated with knowledge of cervical cancer and radiotherapy. This underscores the need for initiatives to enhance HL in tandem with knowledge dissemination.

## Limitations

This study had some limitations. First, our sample might have been biased toward a population that has a greater interest in healthcare, as they voluntarily participated in the survey among the LunaLuna users. As indicated in Supplementary Fig. 1, we observed a decrease in vaccination coverage after age 21, mirroring previous studies, yet the decline was less pronounced, suggesting that our sample might have a greater interest in vaccines than the general population [23]. The high literacy level of our sample might also have introduced selection bias, as individuals with low HL are less likely to use the Internet to seek cancer information [14]. Second, the validity of our questions is a potential issue. Although questions about radiotherapy have been addressed in previous studies, their validity has not been confirmed. Consequently, we cannot unequivocally assert that the results cover the entire knowledge on cervical cancer and radiotherapy. Third, the existence of unmeasured confounders in multiple regression analysis cannot be dismissed. Despite these limitations, it is noteworthy that HL is more strongly associated with knowledge of cervical cancer and radiotherapy than age, cancer diagnosis, or education.

In March 2023, the Japanese Cabinet approved the 4th Basic Plan to Promote Cancer Control Programs. The goal was to “promote cancer control measures that leave no one behind and aim to overcome cancer together with all citizens.” It emphasizes cancer education and the widespread dissemination of cancer-related knowledge as the foundation to establish cancer prevention, treatment, and coexistence. To achieve these objectives, the plan advocates for integrating cancer education programs within school curricula alongside initiatives to enhance cancer knowledge dissemination within workplaces, which is expected to improve cancer knowledge for all generations. A previous Japanese article showed that children improved their knowledge following participation in cancer education programs [24]. Our research has also shown the potential of multimedia, such as informational videos about radiotherapy, to significantly increase confidence in the effectiveness of treatment in the general population [16]. Given the predominance of cervical cancer among individuals in their 30 s and the importance of the HPV vaccine, cancer education in schools is more critical.

Furthermore, decision-making skills are an integral part of health literacy. This is supported by recent evidence showing a correlation between health literacy and decision-making skills in the Japanese population [25]. The recent inclusion of information evaluation and judgment skills in the national curriculum for elementary through high school students, performed by the Ministry of Education, Culture, Sports, Science and Technology in Japan, is a progressive step toward establishing decision-making practices. We argue that, in addition to improving decision-making skills through formal education, a collaborative effort to increase general knowledge and health literacy, mainly through targeted cancer education initiatives, is imperative.

## Conclusion

In this study, we investigated the association between HL and knowledge of cervical cancer and radiotherapy using a web-based survey of women’s healthcare service users. HL showed a stronger association with knowledge of cervical cancer and radiotherapy than sociodemographic background factors such as age, education, and history of cervical cancer screening. Improvement of HL in conjunction with knowledge dissemination may be a crucial measure for promoting cervical cancer prevention and the use of radiotherapy in Japan.

## Supplementary Information

Below is the link to the electronic supplementary material.

Supplementary file1 (PDF 134 KB)

Supplementary file2 (PDF 11 KB)

Supplementary file3 (PDF 60 KB)

Supplementary file4 (PDF 60 KB)

Supplementary file5 (PDF 62 KB)

## Data Availability

The data that support the findings of this study are available from third party. Data are available on request from the corresponding author, M.M. with the permission of MTI Ltd.
