# Supplementary figures and images for: Exploring the Correlation Between Health Literacy and Knowledge of Cervical Cancer and Radiotherapy Among Japanese Women: A Web-Based Survey
Source: J Cancer Educ. 2024 May 29;39(5):530–6. doi: 10.1007/s13187-024-02432-x (PMC11461766; doi:10.1007/s13187-024-02432-x)

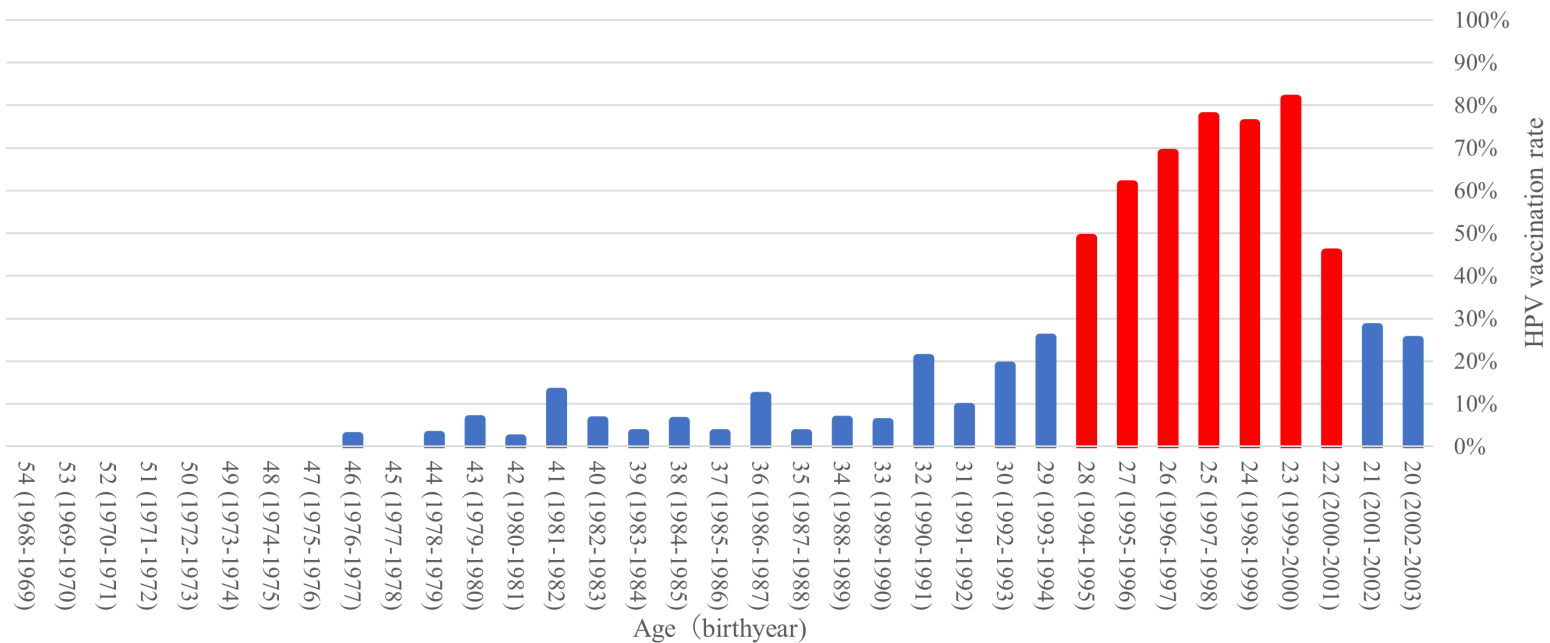

Supplement: Supplementary file 1 — Supplementary file1 (PDF 134 KB) [file 13187_2024_2432_MOESM1_ESM.pdf]
