# Supplementary material for: Exploring the Correlation Between Health Literacy and Knowledge of Cervical Cancer and Radiotherapy Among Japanese Women: A Web-Based Survey
Source: J Cancer Educ. 2024 May 29;39(5):530–6. doi: 10.1007/s13187-024-02432-x (PMC11461766; doi:10.1007/s13187-024-02432-x)
Supplement: Supplementary file 2 — Supplementary file2 (PDF 11 KB) [file 13187_2024_2432_MOESM2_ESM.pdf]

Supplementary Table 1: Reasons for Not Undergoing Cervical Cancer Screening Among Women Aware of It but Haven't Experienced It

	N	%
I feel anxious about the pain caused by the screening.	53	41%
I don't have time for screening.	52	40%
I am concerned about the cost of screening.	36	28%
I can see a doctor whenever I want.	29	22%
I am confident in my health and believe I don't need screening.	24	18%
I often forget to schedule screenings.	24	18%
I fear being diagnosed with cancer.	23	18%
I find the screening location inconvenient.	12	9%
I believe cancer screenings might miss detecting cancer.	5	4%

(Multiple Answers Allowed)
