# Supplementary material for: Exploring the Correlation Between Health Literacy and Knowledge of Cervical Cancer and Radiotherapy Among Japanese Women: A Web-Based Survey
Source: J Cancer Educ. 2024 May 29;39(5):530–6. doi: 10.1007/s13187-024-02432-x (PMC11461766; doi:10.1007/s13187-024-02432-x)
Supplement: Supplementary file 3 — Supplementary file3 (PDF 60 KB) [file 13187_2024_2432_MOESM3_ESM.pdf]

Supplementary Table 2a: Multiple Regression Analysis with the Correct-Answer Rates to Cervical Cancer- and Radiotherapy-Related Questions (Age: 20-29)

| | B | S.E | β | p value |
|---|---|---|---|---|
| Income | 6.72 | 3.13 | 0.12 | 0.032 |
| Education | -8.25 | 2.21 | -0.21 | < 0.01 |
| Health literacy group | 4.16 | 1.41 | 0.16 | < 0.01 |
| $R^2$ | | 0.088 | | |
| *adj.* $R^2$ | | 0.079 | | |

Abbreviations; SE: Standard Error

B: regression coefficient, β: standardized regression coefficient
