# Supplementary material for: Exploring the Correlation Between Health Literacy and Knowledge of Cervical Cancer and Radiotherapy Among Japanese Women: A Web-Based Survey
Source: J Cancer Educ. 2024 May 29;39(5):530–6. doi: 10.1007/s13187-024-02432-x (PMC11461766; doi:10.1007/s13187-024-02432-x)
Supplement: Supplementary file 4 — Supplementary file4 (PDF 60 KB) [file 13187_2024_2432_MOESM4_ESM.pdf]

Supplementary Table 2b: Multiple Regression Analysis with the Correct-Answer Rates to Cervical Cancer- and Radiotherapy-Related Questions (Age: 30-39)

	B	S.E	β	p value
Income	6.61	1.95	0.15	< 0.01
Employment	-4.14	1.68	-0.11	0.037
Cancer history	-9.12	4.60	-0.07	0.048
Health literacy group	5.41	1.02	0.20	< 0.01
R^2		0.101		
adj. R^2		0.096		

Abbreviations; SE: Standard Error

B: regression coefficient, β: standardized regression coefficient
