# Supplementary material for: Exploring the Correlation Between Health Literacy and Knowledge of Cervical Cancer and Radiotherapy Among Japanese Women: A Web-Based Survey
Source: J Cancer Educ. 2024 May 29;39(5):530–6. doi: 10.1007/s13187-024-02432-x (PMC11461766; doi:10.1007/s13187-024-02432-x)
Supplement: Supplementary file 5 — Supplementary file5 (PDF 62 KB) [file 13187_2024_2432_MOESM5_ESM.pdf]

Supplementary Table 2c: Multiple Regression Analysis with the Correct-Answer Rates to Cervical Cancer- and Radiotherapy-Related Questions (Age: 40-)

	B	S.E	β	p value
Income	4.31	1.36	0.09	< 0.01
Education	-5.15	1.56	-0.14	< 0.01
Radiotherapy experience of family	-3.88	1.83	-0.09	0.034
Cervical cancer screening experience	-5.49	1.24	-0.19	< 0.01
Health literacy group	2.42	1.02	0.10	0.018
R^2		0.084		
adj. R^2		0.077		

Abbreviations; SE: Standard Error

B: regression coefficient, β: standardized regression coefficient
